# The Treatment of Gunshot Wound of the Abdomen

**Published:** 1899-10-28

**Authors:** 


### THE TREATMENT OF GUNSHOT WOUND OF THE ABDOMEN.

In discussing the treatment of gunshot wounds of the abdomen at the Portsmouth meeting of the British Medical Association, Colonel Stevenson, professor of military surgery at Netley, laid it down very definitely that when once the fact of penetration of the abdominal cavity is ascertained the only admissible course is to perform laparotomy, to search for the points at which the intestines or the viscera may be wounded, to stop bleeding, and to treat such injuries as may be found by suture, or resection, or by such other method as may seem necessary, but above all things not to stand by hoping that nature will perform a miracle. "When once penetration has been definitely ascertained, diagnosis should be set aside, and treatment should be proceeded with immediately; every half-hour lessens the patient's chance of recovery." So far for general principles. The question is, Can it be done? and in regard to this one must consider the conditions under which, in military surgery, the treatment of the wounded has sometimes to be undertaken. If operative treatment is to be successful it must be undertaken early, and this means that it must be undertaken in field hospitals, mere temporary resting places for wounded men, which may require to be evacuated within a few hours. Again, it may happen that men may have to be left on the ground for a long period before they can be picked up by the bearer companies, and lastly, the surgeon's mind may be influenced by the feeling that the greatest good for the greatest number is the goal for him to aim at. The personnel of the field hospitals is kept as low as possible, and the work done in them during the first hours of battle is performed at extremely high pressure, large numbers of wounded men arriving for treatment almost simultaneously, and the surgeon knows quite well that, as regards the saving of life and limb and suffering, the time spent on one or two abdominal sections might be more profitably expended if devoted to cases less fatally injured. Moreover, beyond all this lies the question how far it is possible to obtain or to maintain the conditions of asepsis which are essential to the success of such operations, and the description given by Major Beevor, R.A.M.C., of the conditions which obtained immediately after the action of Dargai was sufficient to show that almost necessarily fatal as penetrating gunshot wounds of the abdomen are, if left alone, circumstances may and do occur in the course of military operations in which the surgeon is really left no choice in the matter. On the other hand a case quoted from the report by Colonel Whitehead on the Tirah campaign illustrates well what sort of an injury may sometimes be recovered from, even without immediate operation. A native dhoolie bearer was shot in the middle abdominal line, about an inch above the umbilicus. A piece of omentum about the size of a walnut protruded; there had been collapse, which, however, had passed off before he was reached. He was attended to temporarily, and sent on in a dhoolie. Later in the day the force with which he was carried became surrounded by the enemy, and being deserted by the bearers he got out of the dhoolie, and ran away with the rest. He walked three miles to a place of safety, remained out all night without food, in a temperature of 20 degrees of frost, walked into camp eight miles off the next day, and presented himself for treatment; there was then a piece of omentum the size of a fist presenting; it was rendered as aseptic as possible, ligatured, and the pedicle returned into the abdomen, and he made a good recovery. Commenting on the whole discussion, Surgeon-General O'Dwyer concluded that, so far as the reported cases went, those operated on had been in a worse condition than those who had not been operated on. He would say, "Operate by all means provided you can surround the patient with fairly suitable conditions." In fact, it would seem that the question must often be not so much whether operation ought to be done as whether the surroundings are such as to render an operation justifiable.

